# In vivo and in vitro efficacy of the ithmid kohl/zinc-oxide nanoparticles, ithmid kohl/*Aloe vera*, and zinc-oxide nanoparticles/*Aloe vera* for the treatment of bacterial endophthalmitis

**DOI:** 10.1038/s41598-024-66341-1

**Published:** 2024-07-08

**Authors:** Shaimaa Obaid Hasson, Hasanain Kamil Hasan, Sumod Abdul Kadhem Salman, Hawraa K. Judi, Sousan Akrami, Morteza Saki, Maryam Adil Hasan, Durah Fares Hashem

**Affiliations:** 1 Medical Biotechnology Department, College of Biotechnology, Al-Qasim Green University, Babylon, 51013 Iraq; 2 College of Pharmacy, Al-Mustaqbal University, Babylon, 51001 Iraq; 3 Microbiology Department, College of Veterinary Medicine, Al-Qasim Green University, Babylon, 51013 Iraq; 4 Department of Medical Physics, Hilla University College, Babylon, Iraq; 5 Students’ Scientific Research Center (SSRC), Tehran University of Medical Sciences, Tehran, Iran; 6 Department of Microbiology, Faculty of Medicine, Ahvaz Jundishapur University of Medical Sciences, Ahvaz, Iran; 7 Babylon Veterinary Hospital, Agricultural Ministry, Babylon, Iraq

**Keywords:** *Aloe vera*, Bacterial endophthalmitis, Ithmid kohl, ZnONPs, Microbiology, Medical research

## Abstract

The aim of this study was to investigate the efficacy of the ithmid kohl/zinc-oxide nanoparticles (ZnONPs), ithmid kohl/*Aloe vera*, and ZnONPs/*Aloe vera* in the treatment of bacterial endophthalmitis. The endophthalmitis model was prepared by contaminating both eyes of 24 healthy adult male albino rabbits with a clinical isolate of *Klebsiella pneumoniae*. The animals were randomly divided into eight groups (A-H) according to the treatment. Group A received 1 ml of ithmid kohl/ZnONPs ointment, group B received 1 ml of ithmid kohl/*Aloe vera* gel ointment, group C received 1 ml of ZnONPs/*Aloe vera* gel ointment, and groups D, E, and F were treated with 1 ml of ithmid kohl solution (0.5 g/ml in distilled water), 1 ml of ZnONPs (0.5 g/ml) colloidal dispersion, and 1 ml of *Aloe vera* gel, respectively. Group G received 100 μl of a tetracycline antibiotic solution (final concentration: 16 µg/ml), and group H received sterile distilled water (no treatment). In vitro antibacterial activity was evaluated against *K. pneumoniae* using the agar well diffusion. The combination of ithmid kohl/ZnONPs was the most effective formulation for treating endophthalmitis model in infected rabbits within 2 days. In vitro antibacterial assay confirmed the potential of the ithmid kohl/ZnONPs formulation, which had the largest zone of inhibition (31 mm) among the compounds tested. The preparation of the ithmid kohl/ZnONPs formulation and its in vivo experiment in albino rabbits for the treatment of bacterial endophthalmitis was an innovative approach that has shown promise and may potentially serve as a viable alternative in clinical practice.

## Introduction

Bacterial endophthalmitis is an uncommon intraocular infection caused by eye damage, surgery, or hematogenous dissemination of bacteria^1^. Among all types of endophthalmitis, postoperative endophthalmitis (72%), posttraumatic endophthalmitis (20%), and endogenous endophthalmitis (8%) are the most common^2^. As part of the treatment process, the antibiotic susceptibility profiles of the clinically relevant endophthalmitis causing bacteria must be taken into consideration^1^.

Ceftazidime or amikacin are first-line antibiotics for the treatment of endophthalmitis caused by Gram-negative bacteria, while vancomycin is routinely used for the Gram-positive bacteria^1^. However, the global increase in antibiotic resistance has a significant negative impact on the treatment of ocular infections^3^. Moreover, the issue of antibiotic selection for the treatment of eye infection has become increasingly crucial due to the emergence of multidrug-resistant (MDR) bacteria^1,4^.

Ithmid (antimony) kohl (surma), an ancient traditional ophthalmic preparation in the Middle East, has been reported to have antibacterial activity^5^. Due to its ability to stimulate the production of nitric oxide, ithmid kohl has been used in ophthalmology as an antimicrobial and immunostimulating agent. However, due to its composition of heavy metals and other toxic elements, its use has been restricted^5^. Therefore, the fabrication of a new formula of natural kohl in combination with zinc-oxide nanoparticles (ZnONPs) and some medicinal plants has been proposed as an alternative treatment approach for bacterial endophthalmitis. One of these medicinal plants that has shown promising effects in the treatment of various infections in the combination with nanoparticles is *Aloe vera*. Since ancient times, *Aloe vera* has been known for its medicinal properties including anti-inflammatory, antioxidant, and antimicrobial effects^6^. *Aloe vera* contains natural anthraquinones, which play an important role in its antimicrobial properties^7^.

Recent advances have also been made in the treatment and diagnosis of ocular disorders using nanoparticles (NPs)^8,9^. In a recent study by El-Telbany et al.^10^ the efficacy of ZnONPs in the treatment of bacterial keratitis was demonstrated^10^. By interfering with the synthesis of the cell membrane and the cell wall, ZnONPs show significant antimicrobial activity^11^. So far, the antibacterial effects of ithmid kohl in combination with NPs and medicinal herbs have not been well evaluated. There is also, little knowledge about the combination of NPs and *Aloe vera* plant in the treatment of eye infections. The combination of ithmid kohl with these compounds as well as the combination of ZnONPs with *Aloe vera* is likely to be an effective and safe treatment option for bacterial endophthalmitis. Hence, this study aimed to investigate the effectiveness of the ithmid kohl/ZnONPs, ithmid kohl/*Aloe vera*, and ZnONPs/*Aloe vera* for the treatment of bacterial endophthalmitis.

## Materials and methods

### Ethical approval

The Institutional Review Board (IRB) of Al-Qasim Green University, Al-Qasim, Iraq, approved all experimental protocols in this study. All methods were in accordance with ARRIVE's guidelines. The animal experiments in this study were carried out in compliance with the U.K. Animals (Scientific Procedures) Act, 1986, and its guidelines, the ARVO Statement for the Use of Animals in Ophthalmic and Vision Research, and EU Directive 2010/63/EU. The collection and use of the plants were in accordance with all the relevant guidelines. No clinical sample was used to isolate the bacteria in the current study.

### Study design

In this cross-sectional study (February to August 2022), the efficacy of the ithmid kohl/ZnONPs and ithmid kohl/*Aloe vera* in the treatments of bacterial endophthalmitis was investigated in vivo and in vitro.

### Green synthesize of ZnONPs

ZnONPs were green synthesized using an aqueous leaf extract of *Punica granatum* from our previous study by Rais et al.^11^. All the steps of synthesis and characterization of the ZnONPs are included in our previous experiment by Rais et al.^11^. Then the ZnONPs were characterized by UV–Vis spectrophotometer (UV-260, Shimadzu Corp. Tokyo, Japan), Fourier transform infrared (FTIR) spectroscopy (VERTEX-70, Bruker spectroscopy, Optik GmbH Ettlingen, Germany), X-ray diffraction (XRD) (PAN analytical BV, Netherlands), scanning electron microscopy (SEM) (JSM 5600, JEOL, Tokyo, Japan), and transmission electron microscopy (TEM) (Tecnai G220 (FEI) S-Twin 200kv, FEI Co., USA)^11^.

### Preparation of the *Aloe vera* gel

*Aloe vera* plants were prepared and collected from a local house garden in Hillah City, Babylon, Iraq. The fresh plant materials were washed under running tap water, the green sheath of the *Aloe vera* was removed, and the *Aloe vera* gel was extracted with a sharp tool. The gel was then cut into small pieces and put into a blender to mix. Subsequently, it was collected and placed in a clean container (weighing up to 250 g). Finally, the gel was stored in the refrigerator (4 °C) until use.

### Preparation of the ithmid kohl

The antimony stone (500 g) was prepared from a local grocery store in Hillah City, Babylon, Iraq. In next step, antimony stone was broken into small pieces and then ground and made into ithmid kohl powder. The powder was collected and stored in a clean container until use.

### Formulation of the ithmid kohl/ZnONPs ointment

Ointment was initially selected as the formulation since it has the ability to remain on surfaces longer than liquid formulations, and is also easy to rub^12^. To prepare an ointment with a uniform consistency and smooth texture of ithmid kohl/ZnONPs, 2.5 g of ithmid kohl and 2.5 g of ZnONPs were mixed with commercially available Vaseline (up to 100 g). The ointment was transferred to a clean container and stored in the refrigerator (+ 4 °C) until use. The above-mentioned concentrations were randomly selected to carry out a preliminary study^12^.

### Formulation of the ithmid kohl/*Aloe vera* gel ointment

To prepare the ithmid kohl/*Aloe vera* gel ointment, 2.5 g of ithmid kohl and 2.5 g of *Aloe vera* gel were mixed with commercially available Vaseline (up to 100 g). Then, the ointment was transferred to a clean container and stored in the refrigerator (+ 4 °C) until use.

### Formulation of the ZnONPs/*Aloe vera* gel ointment

To prepare ZnONPs/*Aloe vera* gel ointment, 2.5 g of ZnONPs and 2.5 g of *Aloe vera* gel were mixed with commercial Vaseline (up to 100 g). Then, the ointment was transferred to a clean container and stored in the refrigerator (+ 4 °C) until use.

### In vivo experiment

#### Experimental animals

For this study, 24 healthy adult male albino rabbits (1.5–2 kg, aged 1–2 years) were procured from Al-Magmoaa Veterinary Clinic (Iraq). The rabbits were kept in cages and exposed to a 12/12 h light/dark cycle at room temperature, while being provided with food and water^12^. The experiment only started after 1 week to allow the rabbits to acclimatize to the home environment^12^.

#### Creation of the endophthalmitis model

The endophthalmitis model was created by contaminating both eyes of all albino rabbits with a clinical *K. pneumoniae* isolate. This isolate was obtained from a male patient (35 years old) with endophthalmitis infection. The *K. pneumoniae* isolate was confirmed in the microbiology laboratory using standard biochemical differential tests^13^. An intramuscular injection of a mixture of ketamine hydrochloride (50 mg/kg) and xylazine hydrochloride (4 mg/kg) (Sigma Aldrich, USA) was used to induce general anesthesia to perform all examinations. A *K. pneumoniae* suspension with a concentration of 1.0 × 10^5^ CFU/ml was prepared for ocular inoculation. To reduce the intraocular pressure to a satisfactory level, an anterior chamber paracentesis was performed using a 27-gauge needle. Subsequently, 100 µl of the *K. pneumoniae* suspension was injected with a 30-gauge needle 6 mm away from the corneal limbus^14^. After 24 h, the eyes of the rabbits showed signs of endophthalmitis, including hypopyon in the anterior chamber and edema of the conjunctiva^14^. After 2 days, the inflammatory symptoms intensified, and the eyeballs demonstrated retinal necrosis, indicating the establishment of intraocular infection^14^.

#### Animal treatment grouping and evaluation

At the beginning of the experiment, 24 animals were randomly divided into eight groups (A–H) according to the treatment they received (Table 1). After 48 h, during which the endophthalmitis model was established, all groups received the therapeutic combination topically twice daily. Group A received 1 ml of ithmid kohl/ZnONPs ointment, group B received 1 ml of ithmid kohl/*Aloe vera* gel ointment, group C received 1 ml of ZnONPs/*Aloe vera* gel ointment, and groups D, E, and F were treated with 1 ml of ithmid kohl solution (0.5 g/ml in distilled water), 1 ml of ZnONPs (0.5 g/ml) colloidal dispersion, and 1 ml of *Aloe vera* gel, respectively. Group G received 100 μl of tetracycline antibiotic solution (Sigma, USA) (a final concentration of 16 µg/ml) as a standard control and group H received sterile distilled water (no treatment) as an infected control. Clinical examination of the infected eyes of the rabbits was performed daily with a slit-lamp microscope until full healing and photographs were taken. The full healing was considered based on the lack of any signs of infection such as inflammation, pus, swelling, and redness. The analysis of the outcomes of the therapy was carried out in a blinded fashion by a researcher who was unaware of the treatment administered (or not administered). Following the healing process, each group was tracked for 1 month to confirm the absence of infection reoccurrence.

**Table 1:** Grouping of albino rabbits with *Klebsiella pneumoniae*-induced endophthalmitis based on their treatment.

Group	Treatment	Number of tested animals
A	Ithmid kohl/zinc-oxide nanoparticles ointment	3
B	Ithmid kohl/*Aloe vera* gel ointment	3
C	Zinc-oxide nanoparticles/*Aloe vera* gel ointment	3
D	Ithmid kohl	3
E	Zinc-oxide nanoparticles	3
F	*Aloe vera* gel	3
G	Positive control (tetracycline)	3
H	Negative control (sterile distilled water)	3

### In vitro antibacterial activity assay

The antibacterial activity of all compounds and formulations (ithmid kohl, green synthesized ZnONP, *Aloe vera*, ithmid kohl/ZnONPs, ithmid kohl/*Aloe vera*, and ZnONPs/*Aloe vera*) was evaluated using the agar well diffusion method. For this purpose, a *K. pneumoniae* isolate was recovered from one of the examined rabbits with endophthalmitis infection. The *K. pneumoniae* inoculum was prepared with a turbidity corresponding to the McFarland 0.5 standard tube (1.5 × 10^8^ CFU/ml) and then seeded onto a Muller Hinton agar (Merck, Germany) plate using streaking method^15^. Wells of 6 mm diameter were then made using a sterile cork borer and filled with 100 μl of each of the compounds and formulations tested as follows: ithmid kohl solution (0.5 g/ml in distilled water), ZnONPs (0.5 g/ml), *Aloe vera* (2.5% w/v), ithmid kohl/ZnONPs (5.0% w/v), ithmid kohl/*Aloe vera* (5.0% w/v), and ZnONPs/*Aloe vera* (5.0% w/v). The negative control consisted of 100 µl of sterile distilled water and the positive control was a 100 μl of tetracycline antibiotic solution (Sigma, USA) (a final concentration of 16 µg/ml). All plates were incubated at 37 °C for 24 h. After incubation, the inhibition zone diameter (mm) was measured by a ruler^15^.

### Statistical analysis

The difference among studied compounds and the control (tetracycline) was evaluated by GraphPad Prism 9.0.0 software (San Diego, CA, USA) using one-way ANOVA or Kruskal–Wallis test. *P*-value ≤ 0.05 was considered a significant statistical difference.

### Ethics statement

Al-Qasim Green University's Institutional Review Board (IRB), Al-Qasim, Iraq approved all experimental protocols in this study and all methods followed ARRIVE's guidelines. Animal experiments in this study were carried out in compliance with the U.K. Animals (Scientific Procedures) Act, 1986, and its guidelines, the ARVO Statement for the Use of Animals in Ophthalmic and Vision Research, as well as the EU Directive 2010/63/EU.

## Results

### Main characteristics of green synthesized ZnONPs

**Figure 1:**
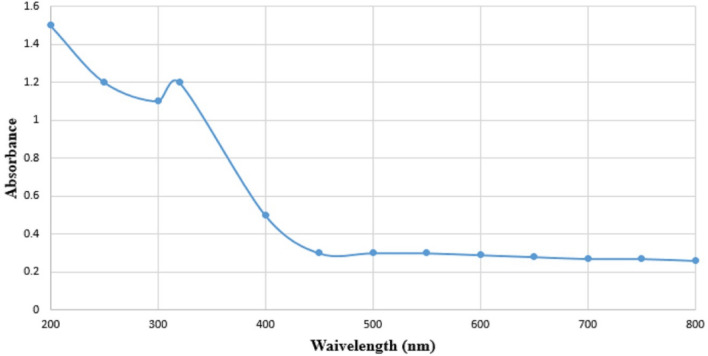
UV–visible analysis of green synthesized zinc-oxide nanoparticles using aqueous leaf extract of *Punica granatum*.

Based on our previous study^11^, the UV–visible spectroscopy revealed a maximum absorption peak (λ) at a wavelength of 310 nm, which is characteristic of the green synthesized ZnONPs (Fig. 1). The FTIR analysis showed strong absorption peaks at 1539.20, 1411.89, 1346.31, 1026.13, 671.23, 613.23, and 563.21 cm^−1^, indicating the presence of O–H, –C≡C, C=O, C–C, C–H, C–O, and C–N stretching, respectively^11^. XRD analysis confirmed the crystalline nature of the green synthesized ZnONPs^11^. SEM and TEM investigations identified ZnONPs with a spherical shape and an average size of 36 nm^11^.

### In vivo observations

**Figure 2:**
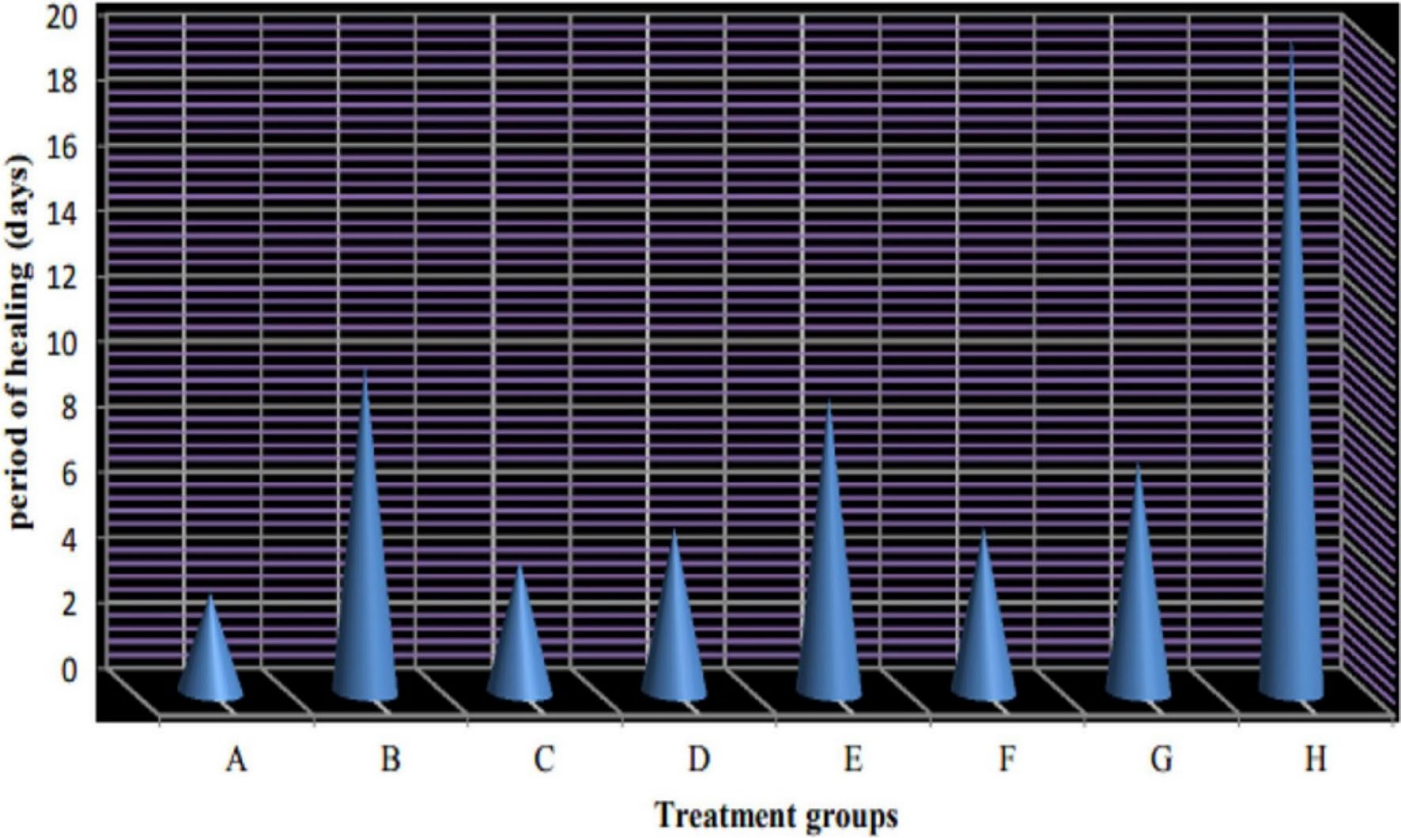
Duration (days) of the healing of endophthalmitis model in albino rabbits. Group A (1 ml of ithmid kohl/zinc-oxide nanoparticles ointment): 2 days; Group B (1 ml of ithmid kohl/*Aloe vera* gel ointment): 9 days; Group C (1 ml of zinc-oxide nanoparticles/*Aloe vera* gel ointment): 3 days; Group D (1 ml of ithmid kohl solution): 4 days; Group E (1 ml of zinc-oxide nanoparticles colloidal dispersion): 8 days; Group F (1 ml of *Aloe vera* gel): 4 days; Group G (100 μl of tetracycline antibiotic solution): 6 days; Group H (no treatment): 19 days.

**Figure 3:**
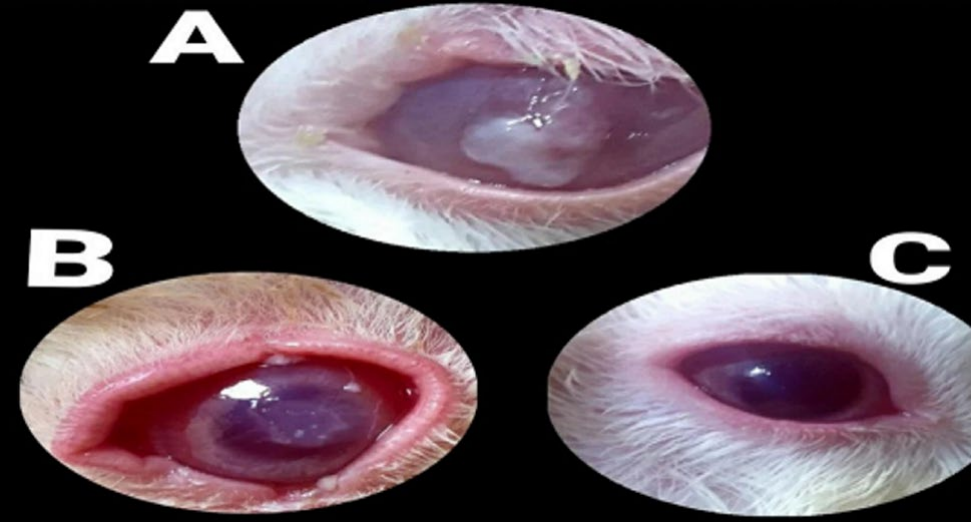
The healing process in group A which received 1 ml of ithmid kohl/zinc-oxide nanoparticles ointment was 2 days. (**A**) day 0, (**B**) day 1, (**C**) day 2.

**Figure 4:**
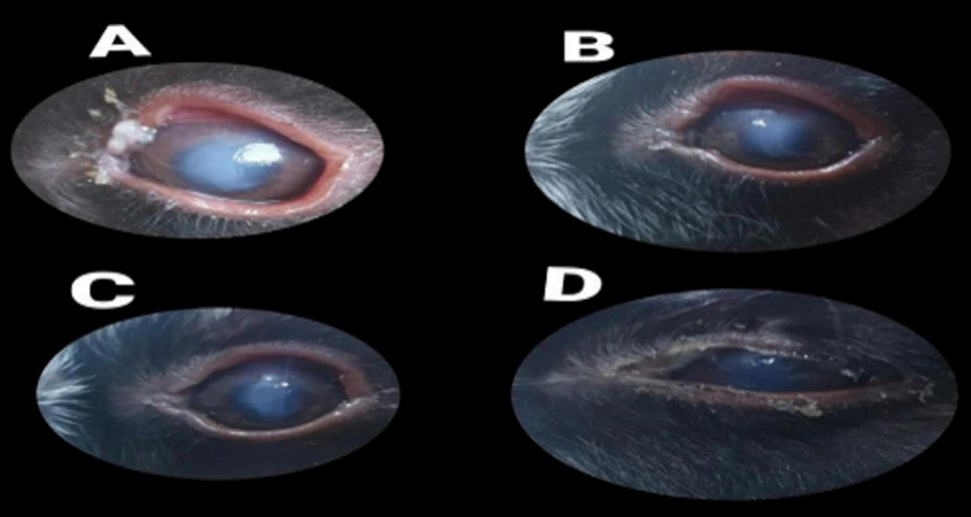
The healing process in group B which received 1 ml of ithmid kohl/*Aloe vera* gel ointment was 9 days. (**A**) day 0, (**B**) day 3, (**C**) day 6, (**D**) day 9.

**Figure 5:**
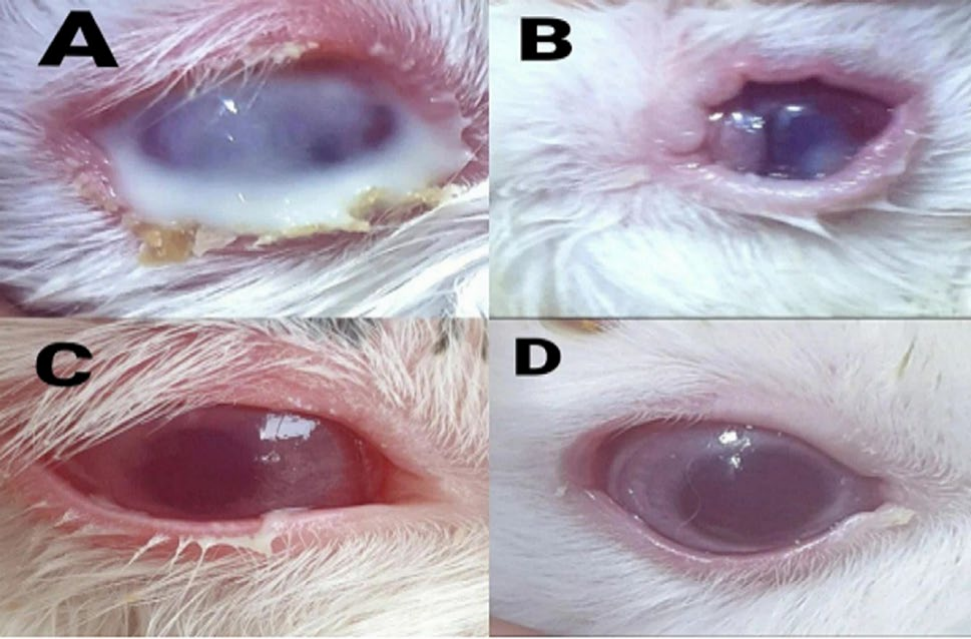
The healing process in group C which received 1 ml of zinc-oxide nanoparticles/*Aloe vera* gel ointment was 3 days. (**A**) day 0, (**B**) day 1, (**C**) day 2, (**D**) day 3.

**Figure 6:**
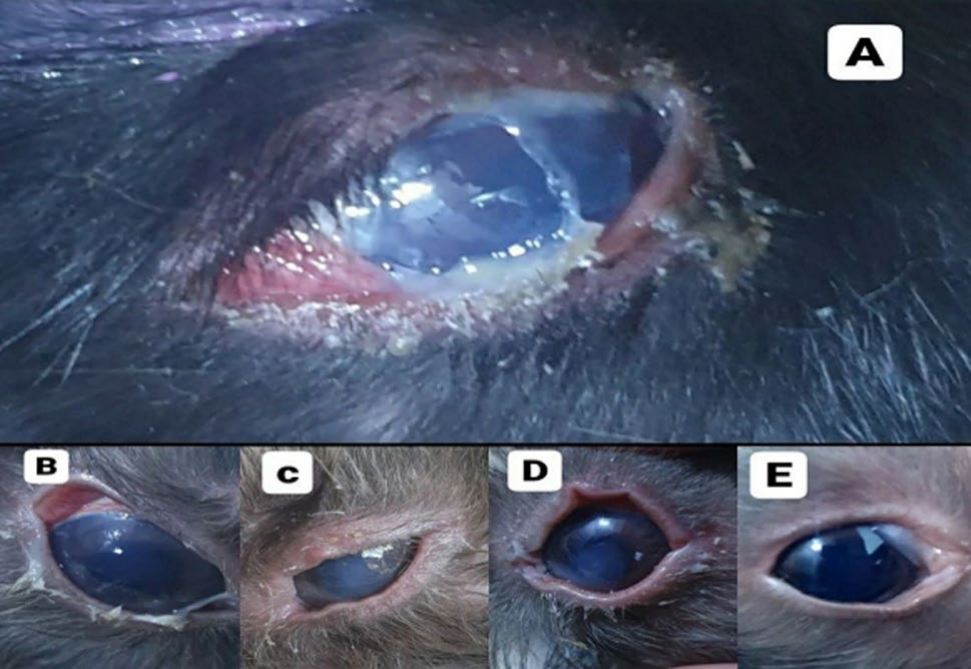
The healing process in group D which received 1 ml of ithmid kohl solution was 4 days. (**A**) day 0, (**B**) day 1, (**C**) day 2, (**D**) day 3, (**E**) day 4.

**Figure 7:**
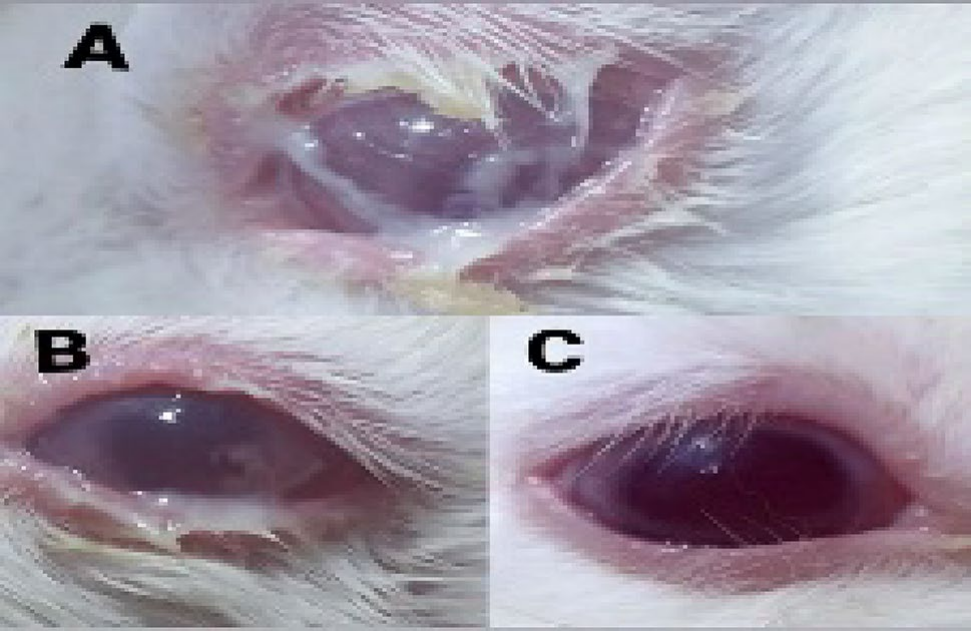
The healing process in group E which received 1 ml of zinc-oxide nanoparticles colloidal dispersion was 8 days. (**A**) day 0, (**B**) day 4, (**C**) day 8.

**Figure 8:**
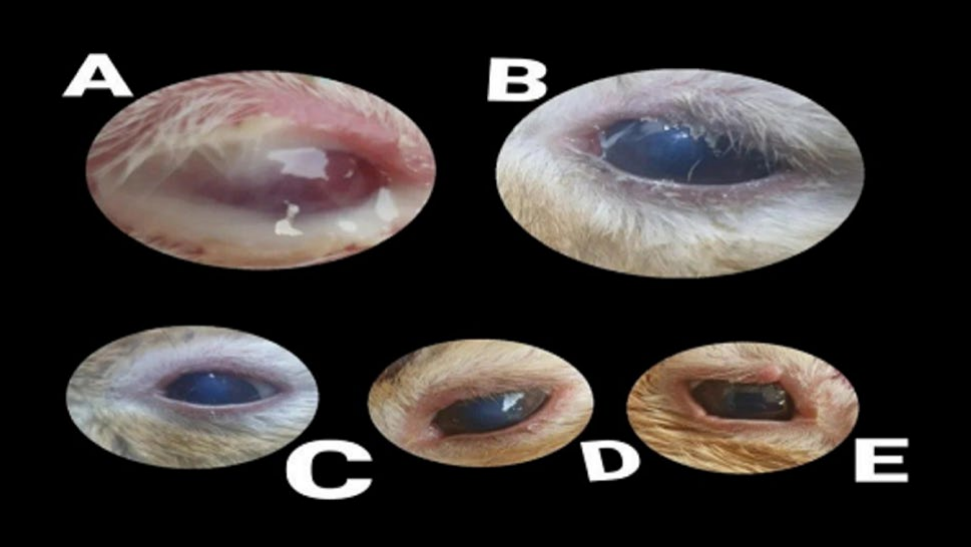
The healing process in group F which received 1 ml of *Aloe vera* gel was 4 days. (**A**) day 0, (**B**) day 1, (**C**) day 2, (**D**) day 3, (**E**) day 4.

**Figure 9:**
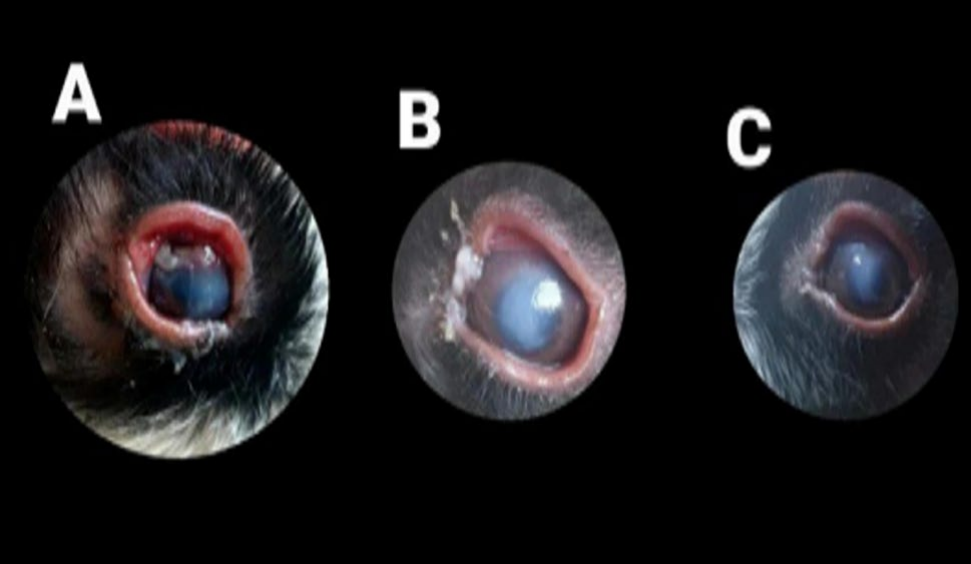
The healing process in group G which received 100 μl of tetracycline antibiotic solution was 6 days. (**A**) day 0, (**B**) day 3, (**C**) day 6.

**Figure 10:**
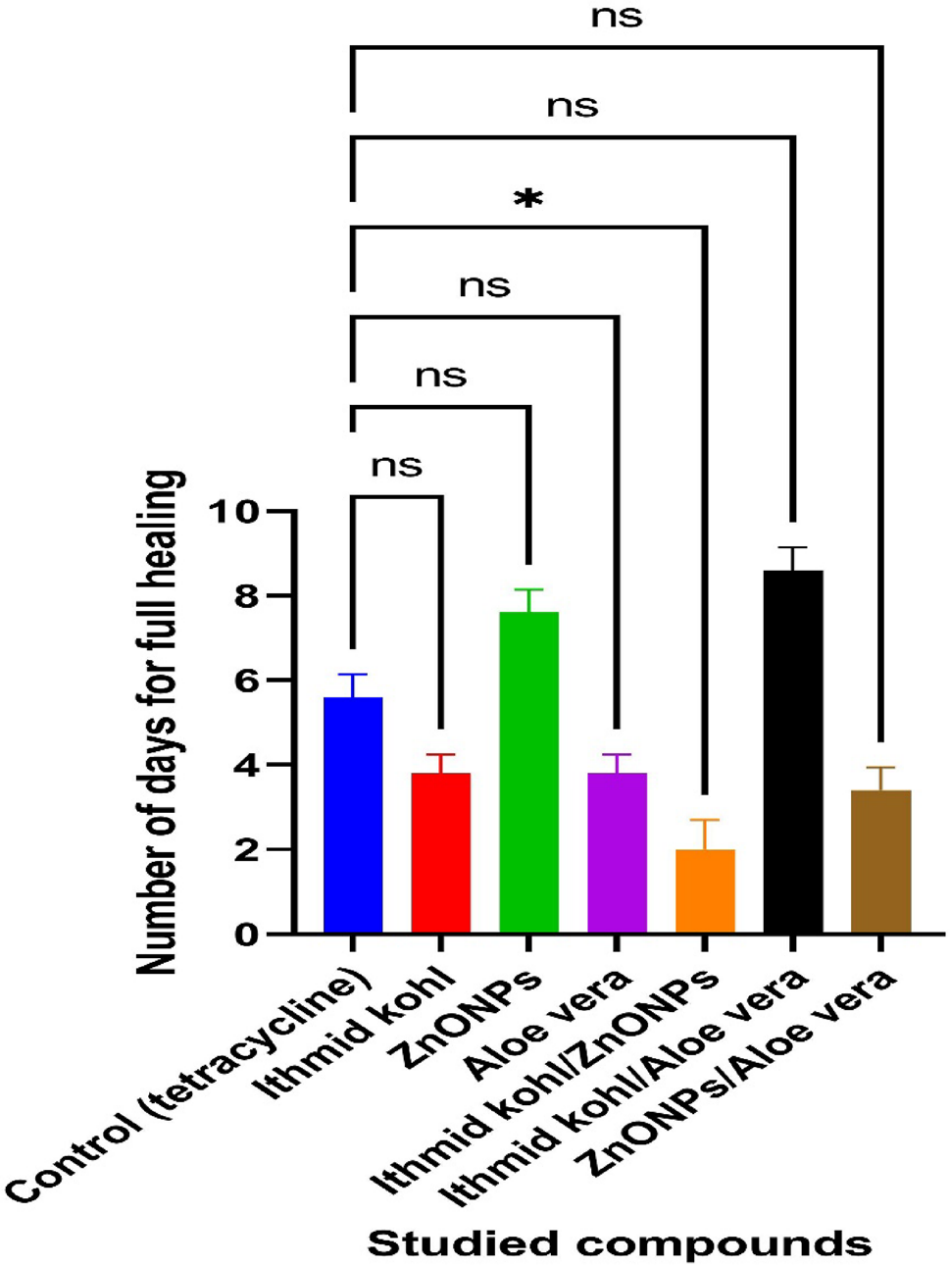
Comparison of studied compounds with control (tetracycline) in the full healing of endophthalmitis model in albino rabbits. *There was a significant difference (*P*-value = 0.002) between the effect of ithmid kohl/ZnONPs ointment and tetracycline antibiotic solution in the full healing process.

The duration (days) of healing of the endophthalmitis model in albino rabbits is shown in Fig. 2. The healing period in group A, which received 1 ml of ithmid kohl/ZnONPs ointment, was 2 days (Fig. 3). The healing time of the group B, which received 1 ml of ithmid kohl/*Aloe vera* gel ointment, was 9 days (Fig. 4). The healing process of group C took 3 days after administration of 1 ml of ZnONPs/*Aloe vera* gel ointment (Fig. 5). The healing of group D occurred over a period of 4 days after the administration of 1 ml of ithmid kohl solution (Fig. 6). Following the application of 1 ml of ZnONPs colloidal dispersion, group E recovered in a span of 8 days (Fig. 7). Group F regained their health over a period of 4 days after the application of 1 ml of *Aloe vera* gel (Fig. 8). Applying 100 μl of tetracycline antibiotic solution to group G resulted in their healing within 6 days (Fig. 9). Finally, auto-healing was observed in group H in a period of 19 days, which was an infected eye model without treatment. The ithmid kohl/ZnONPs ointment caused earlier complete healing of endophthalmitis than the control group, and there was a significant difference (*P*-value = 0.002) between the effect of ithmid kohl/ZnONPs ointment and the tetracycline antibiotic solution in the complete healing of the endophthalmitis model in albino rabbits (Fig. 10).

### In vitro antibacterial property

**Figure 11:**
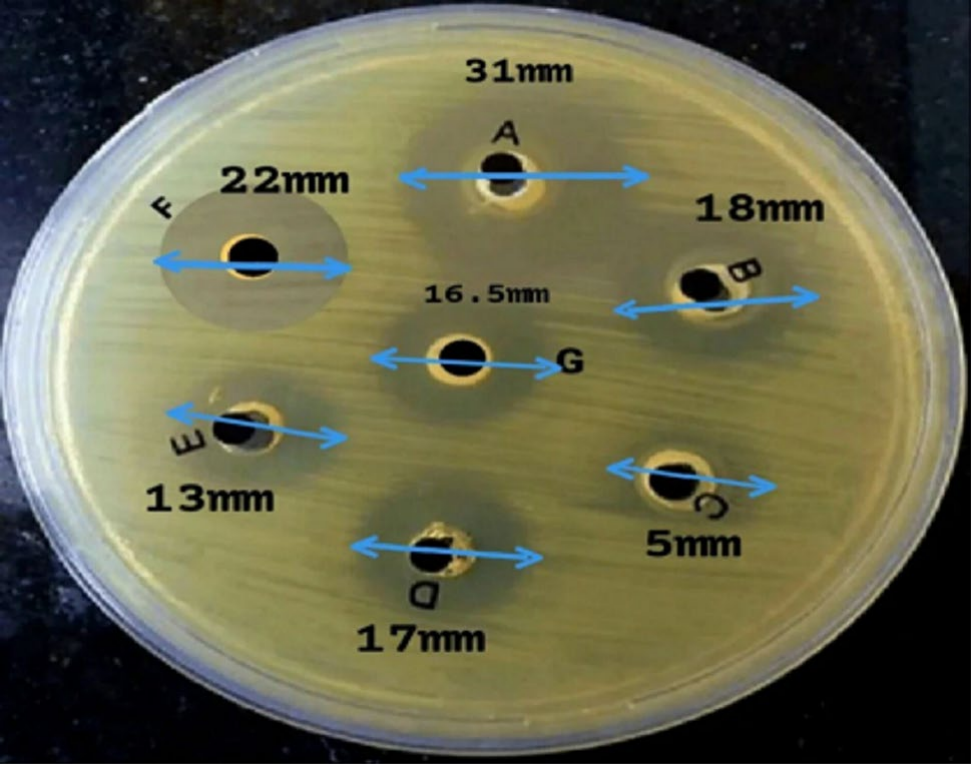
In vitro antibacterial activity of different studied compounds against *Klebsiella pneumoniae* isolate according to inhibition zone diameter (mm). (**A**) ithmid kohl/zinc-oxide nanoparticles (31 mm); (**B**) zinc-oxide nanoparticles/*Aloe vera* gel (18 mm); (**C**) ithmid kohl/*Aloe vera* gel (5 mm); (**D**) ithmid kohl (17 mm); (**E**) zinc-oxide nanoparticles (13 mm); (**F**) tetracycline antibiotic solution (22 mm); (**G**) *Aloe vera* gel (16 mm).

The in vitro antimicrobial effects of the different studied compounds from the strongest to the weakest according to the diameter of the inhibition zone (mm), were as follows: ithmid kohl/ZnONPs (31 mm), ZnONPs*/Aloe vera* gel (18 mm), ithmid kohl (17 mm), *Aloe vera* gel (16 mm), ZnONPs (13 mm), and ithmid kohl/*Aloe vera* gel (5 mm) (Fig. 11). The inhibition zone diameter of the tetracycline antibiotic solution was 22 mm.

## Discussion

Bacterial endophthalmitis is a significant problem in ophthalmology, for which there are various causative factors^16^. The escalating issue of antibiotic resistance makes treatment even more difficult. In this context, research into alternative treatment methods is essential. In this study, the potential in vivo and in vitro effects of a novel formulation of ithmid kohl/ZnONPs, ithmid kohl/*Aloe vera* gel, and ZnONPs*/Aloe vera* gel for the treatment of bacterial endophthalmitis were investigated for the first time.

Based on the observed results, the combination of ithmid kohl/ZnONPs was more effective in treating the endophthalmitis model in infected rabbits than the other studied compounds and was able to cure the disease in a shorter period of time (2 days). Accordingly, this study showed that the addition of ZnONPs to ithmid kohl formula has antimicrobial properties that can prevent the growth of bacteria in the eye. It was also shown that this formula has anti-inflammatory properties that may help reduce inflammation in the eye caused by bacterial infections. It is possible that the combination of ZnONPs in the ithmid kohl formula acted through synergistic effects that enhanced the overall antimicrobial and anti-inflammatory properties, which needs to be further investigated. The in vitro assay substantiated the potential antibacterial properties of the ithmid kohl/ZnONPs formulation, which exhibited the largest zone of inhibition (31 mm) among the studied compounds. Although the in vitro antimicrobial properties of ithmid kohl have been evaluated in some studies^17,18^, there is scarce data on its in vivo antimicrobial efficiency either alone or in combination with nanoparticles.

In a previous study by Buksh et al.^17^, the antimicrobial effects of 20 kohl samples were examined against some important ocular pathogens including *K. pneumoniae*, *Pseudomonas aeruginosa*, *Proteus mirabilis*, *Staphylococcus aureus*, *Staphylococcus epidermidis*, *Candida albicans*, *Candida tropicalis*, *Aspergillus flavus*, and *Mucor* species by the agar well diffusion method. They observed that kohl samples had the greatest effect on *P. mirabilis* and *S. epidermidis*^17^. Moreover, kohl samples showed antifungal properties^17^. In our study, ithmid kohl alone showed a good in vivo antimicrobial effects, which caused endophthalmitis to heal in 4 days. These results were confirmed through in vitro examination by observing the inhibition zone diameter of 17 mm against *K. pneumoniae* isolate. In a previous clinical trial study by Karbassi et al.^19^ from Iran, which was conducted on 30 patients with staphylococcal blepharitis, the efficacy of kohl in the treatment of blepharitis was significantly higher than that of erythromycin ointment. In another study by Al-Kaff et al.^20^ from Saudi Arabia, certain mixtures of kohl showed slight antimicrobial effects against *Proteus*, *Staphylococcus*, and *Streptococcus* strains. One of the reasons for the diverse antimicrobial properties of kohl in the various studies may be the existence of different formulations of this substance in the markets. Antimony sulfide and trisulfide are the main elements of pure kohl^20^. A shiny and dark stone, called “ithmed” in Arabic, is the origin of kohl^20,21^. Kohl may be composed of only ithmed, or with the addition of materials such as almond seeds, menthol, camphor, and charcoal^20,21^. The limited availability of ithmed has led many manufacturers to replace it with galena (lead sulfide), which has nearly the same hue and glossy finish as ithmed. Additionally, charcoal or other vegetable ashes are being included in the mix by some manufacturers^20,21^.

Investigating the chemical composition of kohl can explain its antibacterial and anti-inflammatory properties. An examination of the chemical components of kohl has revealed that the primary elements are zinc, antimony, carbon, sulphur, and iron^19^. The use of kohl leads to an increase in nitric oxide production, which has antimicrobial capabilities. Moreover, sulphur and antimony in the kohl possess antibacterial properties^19^. Also, the anti-inflammatory properties of kohl are due to the presence of zinc-dependent signaling pathways that reduce inflammation^19^.

One of the unexpected results of this study was the weaker antimicrobial properties of the ithmid kohl/*Aloe vera* gel formulation compared to each individual substance and compared to other tested compounds. The logical scientific rationale for this observation was not obvious, and further investigation is needed to clarify why there was a possible antagonism between ithmid kohl and *Aloe vera* gel.

Another important observation of this study was the efficacy of the ZnONPs*/Aloe vera* gel formula in the treatment of bacterial endophthalmitis. This formula treated the endophthalmitis model in 3 days. The in vitro antimicrobial assay showed a zone of inhibition of 18 mm for this formula, which ranked second among the tested compounds. In a study by Al-Ahbabi et al.^22^, the efficacy of the methanolic extract of *Aloe vera* in the treatment of bacterial keratoconjunctivitis in 20 sheep was similar to that of streptomycin-penicillin. Also, this extract showed significant in vitro antibacterial effects against *Moraxella ovis*, *Proteus* species, and *S. aureus* isolates collected from keratoconjunctivitis cases^22^. Although there are various studies on the efficacy of combining NPs with different antibiotics in the treatment of eye infections in animal models^9,23,24^, the effect of NPs/plants combination in the treatment of such infections has not yet been thoroughly investigated. In a previous study by Elkadery et al.^25^, the curative properties of an aqueous extract of *Nigella sativa* in combination with chitosan nanoparticles were proven in an *Acanthamoeba* keratitis model in albino rats. However, they did not investigate the mechanisms involved in this process^25^.

Although the antimicrobial mechanisms of NPs have not yet been precisely identified, the efficacy of NPs in the treatment of ocular infections is attributed to several mechanisms based on the available data^26,27^. In contrast to the inhibition of protein synthesis, RNA polymerase, folate metabolism, and cytoderm by conventional antibiotics, the antimicrobial effect of NPs is attributed to the disruption of nucleic acids, enzymes, and the cytoderm^27^. Also, the majority of NP forms are able to overcome at least one of the common antibiotic resistance mechanisms^26^. In recent years, the rapid spread of antibiotic-resistant pathogens has been the most pressing global public health problem^28–31^. NPs fight microbes through multiple simultaneous mechanisms^26^. The advantage of these simultaneous mechanisms is that, unlike conventional antibiotics, microbes are unable to cause mutations in multiple genes and eventually develop resistance to NPs^26^.

Despite the very good advantages of the compounds tested in this study, it is not inappropriate to point out some undesirable side effects of these substances. The adverse effects of NP exposure depend largely on the physical and chemical properties of the particles and the dosage of exposure^32^. The ocular system can suffer various adverse effects from exposure to NPs, such as abnormal retinal development, retinal disease, lens vacuolation, dry eye syndrome, and conjunctivitis^27,33^. Studies have shown that normal cells are negatively affected by ZnONPs, although their toxicity is milder compared to other metal oxide NPs^34,35^. The breakdown of ZnONPs resulted in an elevation of zinc ions, triggering mitochondrial dysfunction, caspase activation, and cell apoptosis^27,34^. Moreover, due to the high lead content and contamination with pathogenic bacteria and fungi, kohl may have undesirable side effects leading to the spread of eye infections in many people^17^.

### Limitations

It is important to acknowledge the limitations of this study. Although the in vivo and in vitro results were promising, further research is warranted to validate the safety and efficacy of ithmid kohl/ZnONPs and ZnONPs/*Aloe vera* gel formulations in humans. Additionally, a broader spectrum of bacterial strains should be tested to ascertain the efficacy of this new formula against a wider range of pathogens. Finally, there are few studies on the effects of kohl on endophthalmitis, so it is not possible to compare the results.

## Conclusions

The preparation of two new formulations of ithmid kohl/ZnONPs and ZnONPs/*Aloe vera* gel and their in vivo experiment in albino rabbits for the treatment of bacterial endophthalmitis was an innovative approach that showed promise and could potentially serve as a viable alternative in clinical practice. However, further clinical studies are needed to determine their safety and efficacy in humans, to overcome some of the challenges associated with the production and quality control of the products, and to uncover the mechanisms of their antimicrobial effects. Finally, this study contributes to ongoing efforts to explore alternative treatments for bacterial endophthalmitis in the face of escalating antibiotic resistance.

## Data Availability

The data of the current study are available from the corresponding author on reasonable request.
